# Synthesis, molecular modelling and biological significance of *N*-(4-(4-bromophenyl) thiazol-2-yl)-2-chloroacetamide derivatives as prospective antimicrobial and antiproliferative agents

**DOI:** 10.1186/s13065-019-0564-0

**Published:** 2019-04-01

**Authors:** Deepika Sharma, Sanjiv Kumar, Balasubramanian Narasimhan, Kalavathy Ramasamy, Siong Meng Lim, Syed Adnan Ali Shah, Vasudevan Mani

**Affiliations:** 1 Faculty of Pharmaceutical Sciences, Maharshi Dayanand University, Rohtak, 124001 India; 2 Faculty of Pharmacy, Universiti Teknologi MARA (UiTM), 42300 Bandar Puncak Alam, Selangor Malaysia; 3 Collaborative Drug Discovery Research (CDDR) Group, Pharmaceutical Life Sciences Community of Research, Universiti Teknologi MARA (UiTM), 40450 Shah Alam, Selangor Malaysia; 4 Atta-ur-Rahman Institute for Natural Products Discovery (AuRIns), Universiti Teknologi MARA, 42300 Bandar Puncak Alam, Selangor Malaysia; 5 Department of Pharmacology and Toxicology, College of Pharmacy, Qassim University, Buraidah, 51452 Kingdom of Saudi Arabia

**Keywords:** Synthesis, Thiazole derivatives, Antimicrobial activity, Anticancer activity, MCF7, Molecular docking

## Abstract

To combat the antimicrobial and anticancer drug resistance by pathogens and cancerous cells, efforts has been made to study the pharmacological activities of newly synthesized *N*-(4-(4-bromophenyl)thiazol-2-yl)-2-chloroacetamide derivatives. The molecular structures of the synthesized derivatives were confirmed by their physicochemical properties and spectroanalytical data (NMR, IR and elemental). The synthesized compounds were evaluated for their in vitro antimicrobial activity against bacterial (Gram positive and Gram negative) and fungal species using turbidimetric method and anticancer activity against oestrogen receptor positive human breast adenocarcinoma cancer cell line (MCF7) by Sulforhodamine B (SRB) assay. Molecular docking studies were carried out to study the binding mode of active compounds with receptor using Schrodinger *v11.5.* The antimicrobial activity results revealed that compounds **d1**, **d2** and **d3** have promising antimicrobial activity. Anticancer screening results indicated that compounds **d6** and **d7** were found to be the most active ones against breast cancer cell line. Furthermore, the molecular docking study demonstrated that compounds **d1**, **d2**, **d3**, **d6** and **d7** displayed good docking score within binding pocket of the selected PDB ID (1JIJ, 4WMZ and 3ERT) and has the potential to be used as lead compounds for rational drug designing.
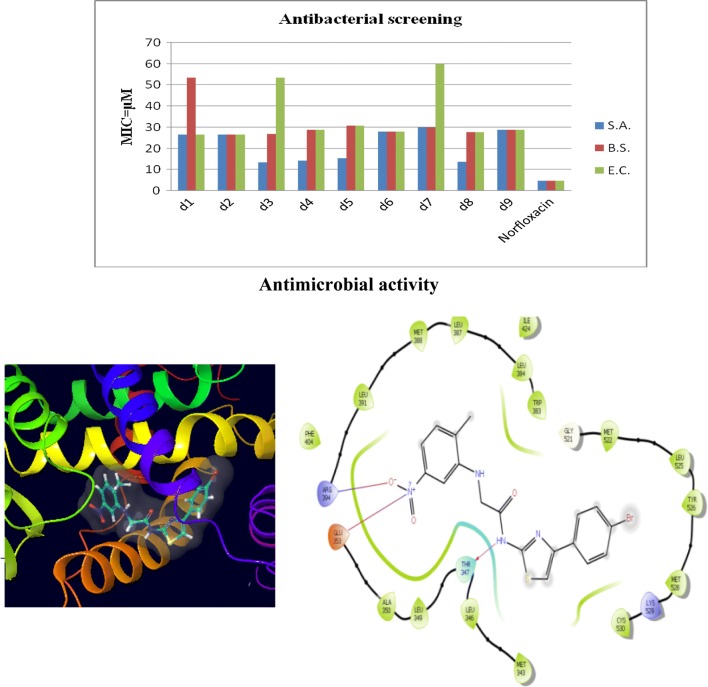

## Introduction

The indiscriminate use of antimicrobial agents has resulted in microbial resistance which has reached on alarming level [1]. This necessitates the need for discovery and development of new molecules from the natural or synthetic sources with novel mode of action to treat microbial infections [2, 3]. Cancer, one of the most dreadful diseases of the current era, is characterized by uncontrolled growth of cells. Its incidence is rising in developed as well as in undeveloped countries. Malignancy is caused by abnormalities in cells, which may be due to inherited genes or caused by outside exposure of the body to chemicals, radiations etc. [4, 5]. To date, there is no ideal cure of cancer. Multidisciplinary scientific approaches are being employed to overcome the various challenges of cancer treatment [6].

**Fig. 1:**
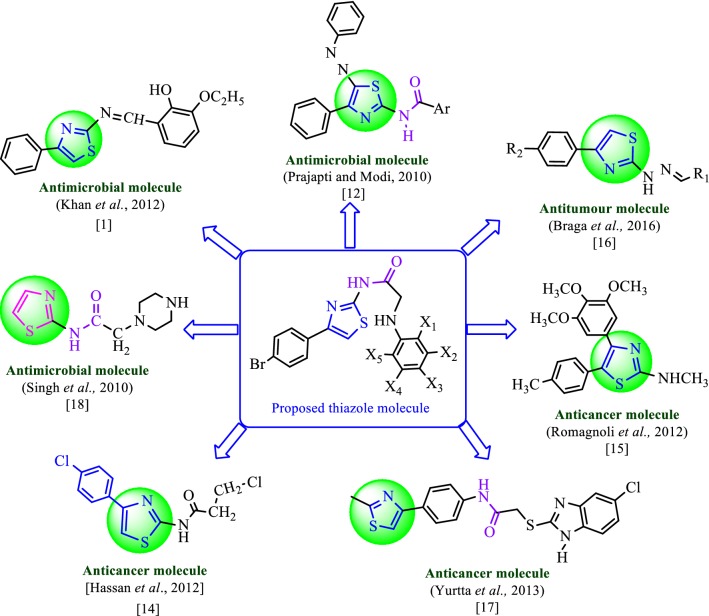
Design of proposed thiazole molecules for antimicrobial and anticancer activity based on literature

The heterocyclic thiazole nucleus reported to have various medicinal properties like anti-inflammatory [7, 8], antibacterial [9, 10], antifungal [11, 12], antitubercular [13] and antitumor [2, 14] activities etc. Thiazole nucleus exhibited its antimicrobial activity by blocking the biosynthesis of certain bacterial lipids and/or by additional mechanisms against various bacterial species [2]. Based on literature reports, it was found that thiazole ring is essential for the antimicrobial [1] and anticancer [15, 16] activities. The presence of electron withdrawing group as *para*-aromatic substituent on 1,3-thiazole nucleus and amide linkage within the molecule can be considered as a useful template for antitumor activity [14, 17]. Prajapti et al. and Singh et al. reported that amino thiazole derivatives with amide linkage exhibit good antibacterial and antifungal activity [12, 18]. Literature based design of the proposed thiazole molecules with antimicrobial and anticancer potential is shown in Fig. 1.

Molecular docking, a cost effective and time saving tool has become an effective and competent approach for rational drug designing. Molecular docking software’s that are available in drug research industry includes AutoDock/Vina, GOLD, FlexX, FRED, DOCK, GLIDE. Docking is an in silico screening technique for the search of suitable ligand–protein complex that fits both energetically and geometrically in the protein’s active site [19]. In light of above facts in continuation of our efforts in search for novel antimicrobial and anticancer agents [20, 21], the present study was undertaken to design, synthesize, molecularly dock and evaluate the biological potentials of *N*-(4-(4-bromophenyl)thiazol-2-yl)-2-chloroacetamide derivatives.

## Experimental part

### Materials and methods

Preliminary material required to carry out the research work was obtained from the commercial sources [Loba Chemie, Pvt Ltd. Mumbai, India Central Drug House (CDH) Pvt. Ltd., New Delhi, India] and used without further purification. Reaction progress was observed by thin layer chromatography making use of commercial silica gel plates (Merck), Silica gel F254 on aluminum sheets, using TLC mobile phase {Chloroform: Toluene (7:3)}. Melting points were determined in open capillary tubes on a Sonar melting point apparatus. Infrared (IR, KBr cm^−1^) spectra were recorded on a Bruker FTIR spectrometer. ^1^H-NMR at 600 MHz and ^13^C-NMR at150 MHz was recorded on Bruker Avance III 600 NMR spectrometer, using appropriate deuterated solvents (DMSO-*d6*). The results are conveyed in parts per million (*δ*, ppm) downfield from tetramethyl silane (internal standard). Proton NMR data are given as multiplicity (s, singlet; d, doublet; t, triplet; m, multiplet) and number of protons. The mass spectral data were recorded on Waters Q-TOF micromass (ESI–MS). Elemental analysis was performed by Perkin-Elmer 2400 C, H and N analyzer.

### General procedure for the synthetic scheme-1

#### Step a: Synthesis of 4-(4-bromophenyl)thiazol-2-amine (Int.-I)

A mixture of *p*-bromoacetophenone (0.1 mol), thiourea (0.2 mol) and iodine (0.1 mol) was taken in a round bottom flask and refluxed for 12 h. The reaction mixture was cooled and washed with diethyl ether to remove the unreacted acetophenone and iodine. The reaction mixture was allowed to cool at room temperature, poured in the solution of ammonium hydroxide and the precipitated crude product was filtered [22].

#### Step b: Synthesis of *N*-(4-(4-bromophenyl)-thiazol-2-yl)-2-chloroacetamide (Int.-II)

Chloro acetyl chloride (0.05 mol) and few drops of triethylacetic acid were added in ethanol (30 ml) and the mixture was stirred on water bath for 10 min. The 4-(4-bromo-phenyl)thiazol-2-amine (prepared in step a) (0.05 mol) was added drop wise to above reaction mixture and refluxed for 2–3 h. The reaction mixture was then cooled and poured into ice cold water and the resultant precipitate was filtered [23].

#### Step c: Synthesis of *N*-(4-(4-bromophenyl)-thiazol-2-yl)-2-(substituted phenylamino) acetamide derivatives (d1-d9)

A mixture of *N*-(4-(4-bromophenyl)thiazol-2-yl)-2-chloracetamide (0.01 mol) and corresponding substituted aniline (0.02 mol) was refluxed for 7–8 h. Then the mixture was cooled, poured into ice cold water and separated product was filtered, washed with water and dried [24].

### Spectral data

#### *4*-*(4*-*Bromophenyl)thiazol*-*2*-*amine* (Int-**I**)

^1^H-NMR *δ*: 7.19–7.56 (m, 4H, ArH), 6.90 (s, 1H, CH of thiazole), 4.12 (s, 2H, –NH_2_); ^13^C-NMR *δ:* 128.4, 127.4, 131.7, 124.3, 122.5, 130.2 (6C of phenyl nucleus), 167.1, 146.4, 98.6 (3C of thiazole); IR: 3113 (C–H str., phenyl nucleus), 1586 (C=C skeletal str., phenyl), 1196 (C–C skeletal str., phenyl), 666 (C–Br str., C_6_H_5_Br), 725 (C–S str., thiazole), 1632 (C=N skeletal str., thiazole), 817 (N–H str., NH_2_), 1265 (C–N str., Ar–NH_2_); Mass: *m/z* 256 [M^+^+1]; CHN: C_9_H_7_BrN_2_S: Theoretical: C, 42.37; H, 2.77; N, 10.98; Found: C, 42.29; H, 2.57; N, 10.78.

#### *N*-*(4*-*(4*-*Bromophenyl)thiazol*-*2*-*yl)*-*2*-*chloroacetamide* (Int-**II**)

^1^H-NMR *δ:* 7.54–7.65 (m, 4H, Ar CH), 8.34 (s, 1H, NH), 7.34 (s, 1H, CH of thiazole), 3.18 (s, 2H, CH_2_–Cl), 7.62 (s, 1H, –CONH); ^13^C-NMR *δ*: 127.6, 130.1, 122.5, 124.2, 127.3, 132.1 (6C of phenyl nucleus), 161.4, 102.5, 151.2 (3C of thiazole); IR: 3444 (C–H str., phenyl nucleus), 1572 (C=C skeletal str., phenyl), 1199 (C–C skeletal str., phenyl), 629 (C–Br str., C_6_H_5_Br), 829 (C–S str., thiazole), 1633 (C=N skeletal str., thiazole), 817 (C–N str., Ar–NH_2_), 1604 (C=O str.), 746 (C–Cl str., CH_2_Cl); Mass: *m/z* 332 [M^+^+1]; CHN: C_9_H_7_BrN_2_S: Theoretical: C, 39.84; H, 2.43; N, 8.45; Found: C, 39.26; H, 2.32; N, 8.41.

#### *N*-*(4*-*(4*-*Bromophenyl)thiazol*-*2*-*yl)*-*2*-*((4*-*chloro*-*3*-*nitrophenyl)amino)acetamide* (**d1**)

^1^H-NMR *δ*: 7.32–7.52 (m, 7H, ArH), 6.84 (s, 1H, CH of thiazole), 7.64 (s, 1H, –CONH) 3.50 (s, 2H, CH_2_), 3.18 (s, 1H, Ar–NH); ^13^C-NMR *δ*: 128.4, 131.1, 121.6, 127.2, 129.3, 131.2, 119.2, 129.5, 113.4, 146.0, 105.4, 143.3 (12C of aromatic nucleus), 163.2, 104.4, 149.5 (3C of thiazole), 164.7 (1C of amide); IR: 3117 (C–H str., phenyl nucleus), 1587 (C=C skeletal str., phenyl), 1671 (C=O str., CONH), 706 (C–Br str., C_6_H_5_Br), 832 (C–S–C str.), 1156 (C–N str., CONH), 1597 (N–H in plane bending, CONH), 1567 (N–O str., NO_2_), 757 (C–Cl str., Ar–Cl); Mass: *m/z* 469 [M^+^+1]; CHN: C_17_H_12_BrClN_4_O_3_S: Theoretical: C, 43.65; H, 2.59; N, 11.98; Found: C, 42.35; H, 2.36; N, 11.67.

#### *N*-*(4*-*(4*-*Bromophenyl)thiazol*-*2*-*yl)*-*2*-*((2*-*chloro*-*4*-*nitrophenyl)amino)acetamide* (**d2**)

^1^H-NMR *δ*: 6.70–7.38 (m, 8H, ArH), 7.90 (s, 1H, CONH), 3.46 (s, 2H, CH_2_), 4.10 (s, 1H, Ar–NH); ^13^C-NMR *δ*: 128.3, 131.0, 122.7, 128.2, 129.3, 132.4, 121.1, 112.8, 148.2, 120.0, 124.3, 134.2 (12C, aromatic nucleus), 164.1, 103.4, 151.2 (3C of thiazole), 166.1 (1C of amide); IR: 3094 (C–H str., phenyl nucleus), 1588 (C=C skeletal str., phenyl), 1633 (C=O str., CONH), 647 (C–Br str., C_6_H_5_Br), 746 (C–S–C str.), 1505 (N–H in plane bending, CONH), 1561 (N–O, NO_2_ str.), 746 (C–Cl str., Ar–Cl); Mass: *m/z* 468 [M^+^+1]; CHN: C_17_H_12_BrClN_4_O_3_S: Theoretical: C, 43.65; H, 2.59; N, 11.98; Found: C, 41.74; H, 2.17; N, 10.98.

#### *2*-*((4*-*Bromophenyl)amino)*-*N*-*(4*-*(4*-*bromophenyl)thiazol*-*2*-*yl)acetamide* (**d3**)

^1^H-NMR *δ*: 6.69–7.70 (m, 8H, Ar–NH), 6.61 (s, 1H, C–H of thiazole), 7.90 (s, 1H, CONH), 3.51 (s, 1H, Ar–NH), 3.33 (s, 2H, CH_2_); ^13^C-NMR *δ*: 128.3, 131.04, 122.7, 128.2, 129.3, 132.4, 131.2, 112.7, 130.2, 142.0, 145.1, 115.0 (12C of aromatic nucleus), 164.1, 103.4, 151.2 (3C of thiazole), 168.2 (1C of amide); IR: 3220 (C–H str., phenyl nucleus), 1585 (C=C skeletal str., phenyl), 1627 (C=O str., CONH), 631 (C–Br str., C_6_H_5_Br), 807 (C–S–C str.), 1505 (N–H in plane bending, CONH), 751 (C–Cl str., Ar–Cl); Mass: *m/z* 469 [M^+^+1]; CHN: C_17_H_13_Br_2_N_3_OS: Theoretical: C, 43.71; H, 2.80; N, 8.99; Found: C, 42.74; H, 2.69; N, 8.76.

#### *N*-*(4*-*(4*-*Bromophenyl)thiazol*-*2*-*yl)*-*2*-*((2*-*nitrophenyl)amino)acetamide* (**d4**)

^1^H-NMR *δ*: 6.63–7.39 (m, 8H, Ar–C–H), 6.63 (s, 1H, C–H of thiazole), 7.40 (s, 1H, CONH), 4.10 (s, 1H, Ar–NH), 3.18 (s, 2H, CH_2_); ^13^C-NMR *δ*: 135.3, 118.1, 124.4, 129.7, 143.3, 110.4, 117.2, 122.5, 129.2, 143.1, 111.4, 131.7 (12C of phenyl nucleus), 163.2, 148.4, 104.5 (3C of thiazole), 166.3 (1C of amide); IR: 3339 (C–H str., phenyl nucleus), 1603 (C=C skeletal str., phenyl), 1622 (C=O str., CONH), 631 (C–Br str., C_6_H_5_Br), 744 (C–S–C str.), 1500 (N–H in plane bending, CONH), 1586 (N–O str. NO_2_); Mass: *m/z* 434 [M^+^+1]; CHN: C_17_H_13_BrN_4_O_3_S: Theoretical: C, 47.12; H, 3.02; N, 12.93; Found: C, 47.10; H, 3.01; N, 12.89.

#### *N*-*(4*-*(4*-*Bromophenyl)thiazol*-*2*-*yl)*-*2*-*((4*-*chlorophenyl)amino)acetamide* (**d5**)

^1^H-NMR *δ*: 7.30–7.70 (m, 8H, Ar–C–H), 6.8 (s, 1H, C–H of thiazole), 7.90 (s, 1H, CONH), 3.90 (s, 1H, Ar–NH), 3.85 (s, 2H, CH_2_); ^13^C-NMR *δ*: 130.1, 122.4, 131.2, 128.6, 131.3, 127.4, 145.2, 113.2, 128.6, 125.7, 131.2, 113.9 (12C of phenyl nucleus), 163.2, 150.1, 104.8 (3C of thiazole), 167.7 (1C of amide); IR: 3116 (C–H str., phenyl nucleus), 1622 (C=C skeletal str., phenyl), 1671 (C=O, CONH), 662 (C–Br str., C_6_H_5_Br), 812 (C–S–C str.), 1567 (N–H in plane bending, CONH), 755 (C–Cl str., Ar–Cl); Mass: *m/z* 434 [M^+^+1]; CHN: C_17_H_12_BrClN_2_OS: Theoretical: C, 47.12; H, 3.02; N, 12.93; Found: C, 47.10; H, 3.01; N,12.89.

#### *N*-*(4*-*(4*-*Bromophenyl)thiazol*-*2*-*yl)*-*2*-*((2*-*methyl*-*5*-*nitrophenyl)amino)acetamide* (**d6**)

^1^H-NMR *δ*: 7.15–7.47 (m, 7H, Ar–H), 7.18 (s, 1H, C–H of thiazole), 7.59 (s, 1H, CONH), 3.17 (s, 1H, CH_2_), 4.00 (s, 1H, Ar–NH), 2.36 (s, 3H, CH_3_); ^13^C-NMR *δ*: 131.2, 121.3, 132.0, 126.8, 129.1, 126.3, 143.1, 127.2, 129.4, 121.7, 141.3, 104.4 (12C of phenyl nucleus), 162.2, 147.1, 103.2 (3C of thiazole), 165.8 (1C of amide); IR: 3126 (C–H str., phenyl nucleus), 1596 (C=C skeletal str., phenyl), 1630 (C=O str., CONH), 647 (C–Br str., C_6_H_5_Br), 813 (C–S–C str.), 1543 (N–H in plane bending, CONH), 1454 (N–O str., NO_2_); Mass: *m/z* 448 [M^+^+1]; CHN: C_18_H_15_BrN_4_O_3_S: Theoretical: C, 48.33; H, 3.38; N, 12.53; Found: C, 48.21; H, 3.30; N, 12.48.

#### *N*-*(4*-*(4*-*Bromophenyl)thiazol*-*2*-*yl)*-*2*-*((3*-*nitrophenyl)amino)acetamide* (**d7**)

^1^H-NMR *δ*: 6.96–7.46 (m, 8H, ArH), 5.79 (s, 1H, C–H of thiazole), 7.48 (s, 1H, CONH), 3.51 (s, 1H, CH_2_), 4.14 (s, 1H, Ar–NH), 3.51 (s, 3H, CH_3_); ^13^C-NMR *δ*: 122.3, 130.2, 126.2, 129.1, 132.0, 125.4, 147.1, 128.2, 105.4, 117.9, 147.3, 111.4 (12C of phenyl nucleus), 163.4, 146.5, 104.0 (3C of thiazole), 165.8 (1C of amide); IR: 3070 (C–H str., phenyl nucleus), 1471 (C=C skeletal str., phenyl), 1614 (C=O, CONH), 1590 (N–H in plane bending, CONH), 678 (C–Br str., C_6_H_5_Br), 752 (C–S–C str.), 1340 (C–N str., CONH), 1570 (N–O, NO_2_ str.); Mass: *m/z* 419 [M^+^+1]; CHN: C_17_H_12_BrN_3_O_3_S: Theoretical: C, 47.12; H, 3.02; N, 12.93; Calculated: C, 47.06; H, 3.01; N, 12.78.

#### *N*-*(4*-*(4*-*Bromophenyl)thiazol*-*2*-*yl)*-*2*-*((4*-*chloro*-*2*-*nitrophenyl)amino)acetamide* (**d8**)

^1^H-NMR *δ*: 7.07–7.44 (m, 7H, ArH), 7.05 (s, 1H, C–H of thiazole), 7.54 (s, 1H, CONH), 3.18 (s, 2H, CH_2_), 3.38 (s, 1H, Ar–NH); ^13^C-NMR *δ*: 122.4, 131.9, 130.3, 127.4, 130.1, 124.2, 121.1, 125.4, 128.4, 141.6, 114.2, 132.5 (12C of phenyl nucleus), 161.5, 148.2, 102.1 (3C of thiazole), 167.8 (1C of amide); IR: 3095 (C–H str., phenyl nucleus), 1454 (C=C skeletal str., phenyl), 1633 (C=O str., CONH), 647 (C–Br str., C_6_H_5_Br), 763 (C–S–C str.), 1602 (N–H in plane bending, CONH), 1560 (N–O str., NO_2_), 722 (C–Cl str., Ar–Cl); Mass: *m/z* 453 [M^+^+1]; CHN: C_17_H_11_BrClN_3_O_3_S: Theoretical: C, 43.65; H, 2.59; N, 11.98; Found: C, 43.54; H, 2.61; N, 11.76.

#### *N*-*(4*-*(4*-*Bromophenyl)thiazol*-*2*-*yl)*-*2*-*((2*-*methyl*-*4*-*nitrophenyl)amino)acetamide* (**d9**)

^1^H-NMR *δ*: 6.64–7.64 (m, 8H, Ar–H), 6.66 (s, 1H, CH of thiazole), 7.85 (s, 1H, CONH), 3.43 (s, 2H, CH_2_), 4.15 (s, 1H, Ar–NH), 2.52 (s, 3H, CH_3_); ^13^C-NMR *δ*: 129.3, 122.8, 130.1, 123.7, 130.1, 128.4, 135.4, 123.2, 150.7, 112.1, 118.3, 126.3 (12C of aromatic nucleus), 161.4, 145.2, 106.7 (3C of thiazole), 162.8 (1C of amide); IR: 3125 (C–H str., phenyl nucleus), 1500 (C=C skeletal str., phenyl), 1672 (C=O str., CONH), 685 (C–Br str., C_6_H_5_Br), 722 (C–S–C str.), 1599 (N–H in plane bending, CONH), 1561 (N–O str., NO_2_ str.); Mass: *m/z* 453 [M^+^+1]; CHN: C_18_H_14_BrN_3_O_3_S: Theoretical: C, 48.33; H, 3.38; N, 12.53; Found: C, 48.17; H, 3.32; N, 12.51.

### Antimicrobial evaluation (in vitro)

The antimicrobial activity of the synthesized compounds of *N*-(4-(4-bromophenyl) thiazol-2-yl)-2-chloroacetamide was evaluated against Gram positive bacteria [*Staphylococcus aureus* (MTCC3160) and *Bacillus subtilis* (MTCC441)], Gram negative bacterium *Escherichia coli* (MTCC443) and fungal strains- *Aspergillus niger* (MTCC281)*; Candida albicans* (MTCC227) by tube dilution method. The stock solution was prepared for the test compounds (**d1**–**d9**) and for the standard drugs (norfloxacin and fluconazole) in acetone to get a concentration of 100 μg/ml and this stock solution was further serially tube diluted [25]. Dilution of test and standard compounds were prepared with double strength nutrient broth-I.P (antibacterial) and sabouraud dextrose broth-I.P (antifungal). The samples were incubated at 37 ± 1 °C for 24 h (bacteria), 25 ± 1 °C for 7 days (*A. niger* and *C. albicans*), respectively and results were recorded in terms of MIC.

### Anticancer evaluation (in vitro)

The anticancer screening of synthesized *N*-(4-(4-bromophenyl)-thiazol-2-yl)-2-chloroacetamide derivatives was conducted against the oestrogen receptor positive human breast adenocarcinoma, MCF7, in comparison to a standard drug (5-fluorouracil) using the SRB assay [26]. Briefly, MCF7 cell was exposed to the compounds for 72 h. Treated cell was being fixed with trichloroacetic acid and then stained with 0.4% (*w/v*) SRB in 1% acetic acid. Unbound dye was removed by five washes with 1% acetic acid solution. Protein-bound dye was solubilized with 10 mM Tris base prior to reading of optical density using a computer-interfaced, 96-well microtiter plate reader. The anticancer activity results were expressed as mean IC_50_ value of at least triplicates.

### Molecular docking study

The selected target proteins (PDB ID: 3ERT, 4WMZ and 1JIJ) required for molecular docking studies were obtained from the RCSB Protein data bank (http://www.rcsb.org/pdb/home/home.do). The selected PDB file was prepared for the molecular docking study using Protein Preparation Wizard (preprocessed, optimized and minimized). A grid is generated around the co crystallized ligand so that it can be excluded and new compounds can be attached to the same active site to study their interactions with receptor. The molecular structures of compounds that are to be docked must be in good representations of as they would appear in a protein–ligand complex. LigPrep module of Schrodinger *v11.5* was used to prepare the ligand (compounds) for docking in Maestro format. The prepared ligand and receptor are docked using extra precision (XP). XP module docked the compounds with better precision and accuracy. The XP parameters like docking score, glide energy and glide emodel value were calculated within the Schrodinger *v11.5* [27–30].

## Results and discussion

### Chemistry

**Scheme 1:**
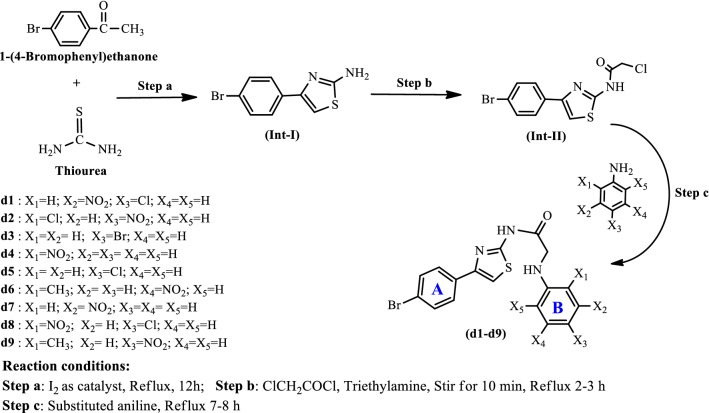
For the synthesis of *N*-(4-(4-bromophenyl)thiazol-2-yl)-2-chloroacetamide derivatives

Syntheses of the intermediate and target derivatives (**d1**–**d9**) were carried out as stated in Scheme 1. Initially, *p*-bromoacetophenone and thiourea were reacted in the presence of catalyst iodine to afford the intermediate 4-(4-bromophenyl) thiazol-2-amine (Step a, Int-**I**). The Int-**I** was further reacted with chloroacetyl chloride to generate (Int-**II**). The Int-**II** was then reacted with corresponding substituted aniline to yield the target compounds (**d1**–**d9**). The molecular structures of the synthesized compounds were confirmed by physicochemical properties (Table 1) and spectral characteristics (IR, NMR, elemental analysis and Mass spectra). The characteristic IR band at 817 cm^−1^ and 666 cm^−1^ indicated the presence of N–H str. of NH_2_ and C–Br str. of C_6_H_5_Br, respectively in Int-**I** and **II**. The IR stretch present at 725 cm^−1^ and 1632 cm^−1^ showed the C–S and C=N linkage, respectively. Therefore, these linkages indicated the existence of thiazole nucleus within the structure of the Int-**I** and **II**. The presence of CONH linkage in int-**II** was confirmed by the presence of C=O group within the range of 1680–1630 cm^−1^ and N–H in plane bending lie within the range of 1500–1640 cm^−1^. The occurrence of band at 3070–3444 cm^−1^ and 1505–1596 cm^−1^ indicated the presence of C–H skeletal and C=C skeletal structure, respectively within the phenyl nucleus. The stretch band present at 1454–1587 cm^−1^ showed the presence of nitro group within the compounds (**d1**, **d2**, **d4** and **d6**–**d9**). The molecular structures of synthesized compounds were further confirmed by ^1^H NMR spectral data. The ^1^H-NMR spectrum of int-**I** showed singlet at 6.9 δ ppm, indicating the presence of NH_2_ group. The ^1^H-NMR spectra of synthesized derivatives displayed multiplet at 6.939–7.52 δ ppm, indicating the aromatic C–H linkage. The presence of singlet at 7.4–7.902 δ ppm indicated the CONH connectivity, confirming of the presence of amide linkage within the synthesized derivatives. All compounds showed singlet at 6.9–7.80 δ ppm which were due to the existence of C–H in thiazole ring. ^13^C-NMR spectra of the thiazole derivatives was recorded in DMSO-*d*_6_ and displayed the fine conformity of their proposed molecular structure i.e. the carbon atoms of phenyl nucleus appeared at 119.2, 127.6, 128.4, 131.2 143.32 δ ppm, carbon atoms of thiazole appearance and carbon of CONH group appeared at 167.6 δ ppm. Elemental analysis of the thiazole derivatives was within the limits of ± 0.5% of the theoretical results.

**Table 1:** Physicochemical characteristics of the synthesized compounds (**d1**–**d9**)

S. No.	Compounds	Molecular formula	M. Wt.	M.P. (^o^C)	R_*f*_ value^a^	% Yield
1.		C_17_H_12_BrClN_4_O_3_S	468	105–107	0.36	73
2.		C_17_H_12_BrClN_4_O_3_S	468	88–90	0.26	68
3.		C_17_H_13_Br_2_N_3_OS	467	118–120	0.41	71
4.		C_17_H_13_BrN_4_O_3_S	433	103–105	0.24	68
5.		C_17_H_13_BrClN_3_OS	407	118–120	0.36	87
6.		C_18_H_15_BrN_4_O_3_S	447	75–77	0.27	76
7.		C_17_H_13_BrN_4_O_3_S	418	108–110	0.32	66
8.		C_17_H_12_BrClN_4_O_3_S	452	78–80	0.22	65
9.		C_18_H_15_BrN_4_O_3_S	432	110–112	0.41	77

^**a**^TLC mobile phase: chloroform: toluene (7:3)

### Antimicrobial activity

**Fig. 2:**
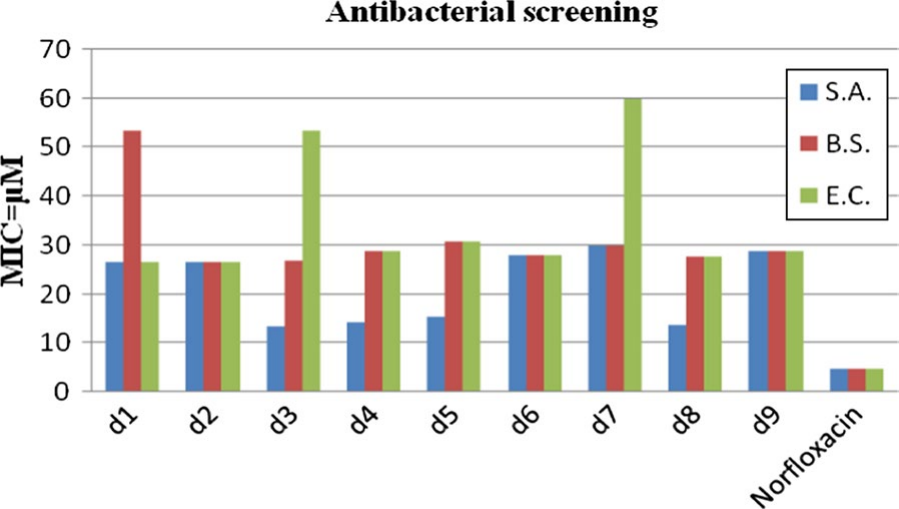
Graphical representation of antibacterial activity of synthesized compounds

**Fig. 3:**
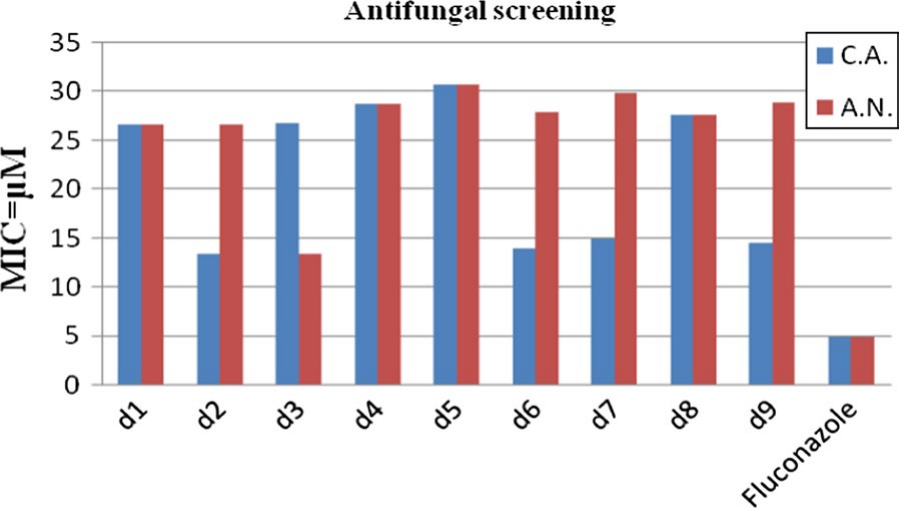
Graphical representation of antifungal activity of synthesized compounds

Antimicrobial screening results indicated that compound **d3** (MIC_sa_ = 13.4 µM) was exhibited promising activity against *S. aureus.* Compound **d1**, on the other hand, was found to be most active against *E. coli* (MIC_ec_ = 26.7 µM). Compound **d2** was found to be the active one against *E. coli* (MIC_ec_ = 26.7 µM) and *B. subtilis* (MIC_bs_ = 26.7 µM), respectively. Antifungal activity results demonstrated that compound **d2** (MIC_ca_ = 13.4 µM) and compound **d3** (MIC_an_ = 13.4 µM) were found to be most active against *C. albicans* and *A. niger,* respectively. The results of antibacterial and antifungal evaluation were expressed as minimum inhibitory concentration (MIC) (Table 2, Figs. 2 and 3).

**Table 2:** Antimicrobial and anticancer activities of synthesized compounds (**d1**–**d9**)

Compounds	Antimicrobial Screening (MIC = µM)					*IC_50_ = µM
	Microbial species					
	Bacterial			Fungal		Cancer cell line
	*S.A.*	*B.S.*	*E.C.*	*C.A.*	*A.N.*	MCF7
**d1**	26.7	53.5	26.7	26.7	26.7	55.6
**d2**	26.7	26.7	26.7	13.4	26.7	76.9
**d3**	13.4	26.8	53.5	26.8	13.4	171.3
**d4**	14.4	28.8	28.8	28.8	28.8	101.6
**d5**	15.3	30.7	30.7	30.7	30.7	132.6
**d6**	27.9	27.9	27.9	14.0	27.9	38.0
**d7**	29.9	29.9	59.8	14.9	29.9	40.6
**d8**	13.8	27.6	27.6	27.6	27.6	44.2
**d9**	28.9	28.9	28.9	14.5	28.9	76.3
**Norfloxacin**	4.7	4.7	4.7	–	–	–
**Fluconazole**	–	–	–	5.0	5.0	–
**5-Fluorouracil**	–	–	–	–	–	5.2

*IC_50_ is the concentration required to kill 50% of cell population

*S.A.*, *Staphylococcus aureus* (MTCC3160); *B.S.*, *Bacillus subtilis* (MTCC441); *E.C.*, *Escherichia coli* (MTCC443); *C.A.*, *Candida albicans* (MTCC227); *A.N.*, *Aspergillus niger* (MTCC281)

### Anticancer activity

**Fig. 4:**
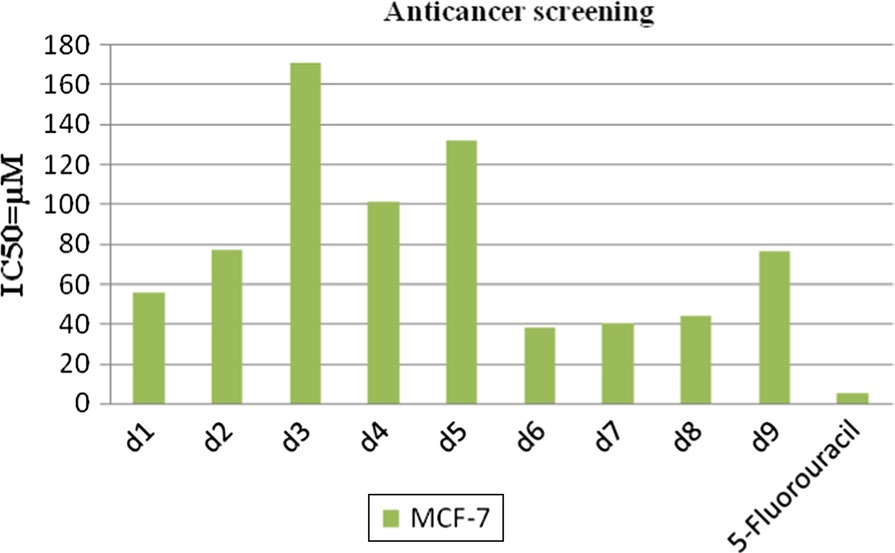
Graphical representation of anticancer activity of synthesized compounds

Anticancer activity results demonstrated that compounds **d6** (IC_50_ = 38.0 µM) and **d7** (IC_50_ = 40.6 µM) were the two most active compounds against MCF7 cancer cell line (Table 2 and Fig. 4). However, the standard anticancer drug, 5-fluorouracil (IC_50_ = 5.2 µM), was more potent when compared to compounds **d6** and **d7**. Compounds **d6** and **d7** may be used as lead molecule for further development of novel anticancer agents.

### Molecular docking

**Fig. 5:**
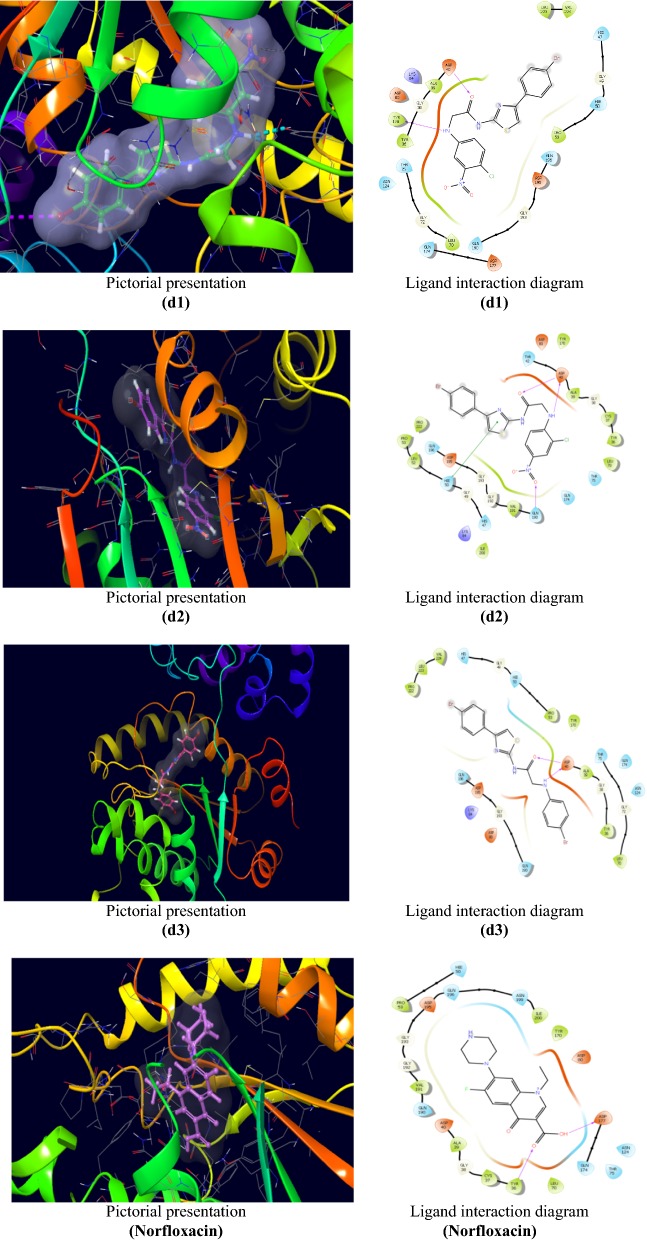
Pictorial presentation (3D) and Ligand interaction diagram (2D) of compounds **(d1** to **d3)** and norfloxacin

**Fig. 6:**
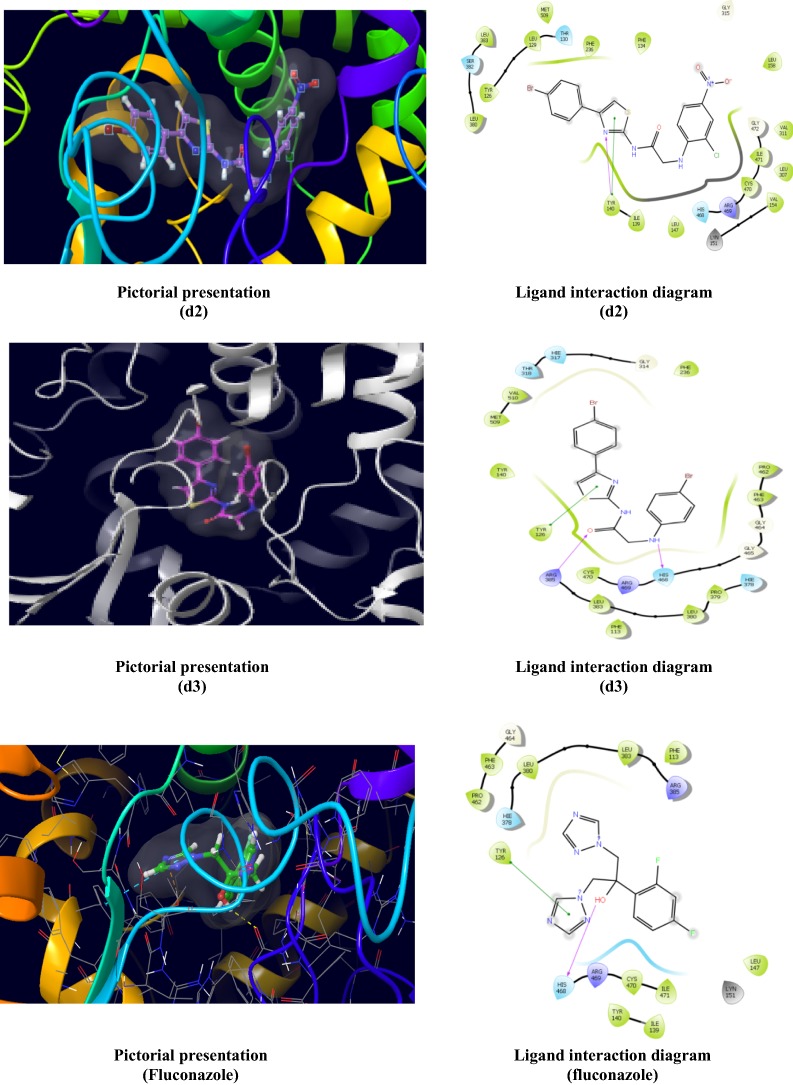
Pictorial presentation (3D) and Ligand interaction diagram (2D) of antifungal compounds (**d2** and **d3**) and fluconazole

Molecular docking was performed to study the binding mode of the synthesized *N*-(4-(4-bromophenyl)thiazol-2-yl)-2-chloroacetamide derivatives with their respective receptors. The PDB files required was identified through literature survey. Docking studies of the most active compounds were carried out using GLIDE module of docking software Schrodinger *v11.5*. Docking score values were used to rank the conformations of these ligand-receptor complexes. Molecular docking study of the most active antibacterial compounds, **d1**, **d2** and **d3** and standard drug (norfloxacin) was done in the active sites of topoisomerse II (PDB ID:1JIJ) obtained from the protein data bank (Table 3). The ligand interaction diagram (2D) and pictorial presentation (3D) of docked compounds and standard drug are as shown in Fig. 5. The 2D ligand interaction diagrammatic view depicted that nitrogen and oxygen atoms of amide nucleus of compounds, **d2** and **d3** and oxygen atom of **d1** formed H-bond with Asp40 amino acid residue. In compound **d2** an additional H-bond was generated with the oxygen atom of nitro group with Gln190 amino acid residue. Molecular docking study of the most active antifungal compounds, **d2** and **d3** and standard drug (fluconazole) was done in the active sites of lantosterol alpha demethylase (PDB:4WMZ) obtained from the protein data bank (Table 4). The ligand interaction diagram (2D) and pictorial presentation (3D) of docked compounds and standard drug are as shown in Fig. 6. The nitrogen atom of compound **d2** showed H-bond interaction with Tyr140 amino acid residue. The nitrogen and oxygen atoms of compound **d3** showed H-bond interaction with the amino acid His468 and Arg385, respectively.

**Table 3:** Docking results of most active antibacterial compounds (**d1**, **d2** and **d3**) with standard drug

Compounds	Docking score	Glide energy (kcal/mol)	Interacting amino acid residues
**d1**	− 5.126	− 62.761	Asp40, Ala39, Gly38, Tyr36, Tyr170, Thr75, Asn124, Gly72, Leu70
**d2**	− 5.403	− 58.496	Thr42, Asp40, Ala39, Gly38, Cys37, Tyr36, Gln190, Val191, Gln192, Gly193, Hie50
**d3**	− 4.806	− 55.908	Tyr36, Gly38, Ala39, Asp40, Pro53, Hie50, Gly49, Gln190, Gly193, Asp195, Gln196
**Norfloxacin**	− 6.18	− 53.349	Asp177, Glu174, Leu70, Tyr36, Cys37, Gly38, Ala39, Asp40, Gln190, Val191, Gly192

**Table 4:** Docking results of most active antifungal compounds (**d2** and **d3**) with standard drug

Compounds	Docking score	Glide energy (kcal/mol)	Interacting amino acid residues
**d2**	− 7.206	− 51.603	Phe236, Thr130, Leu129, Tyr126, Tyr140, Ile139, Leu147, His468, Arg469, Cys470
**d3**	− 8.053	− 50.625	Tyr126, Arg385, Cys470, Arg469, His468, Gly465, Gly464, Phe463, Pro462
**Fluconazole**	− 5.847	− 40.932	His468, Arg469, Cys470, Ile471, Tyr126, Hie378, Leu380, Leu383, Arg385

**Fig. 7:**
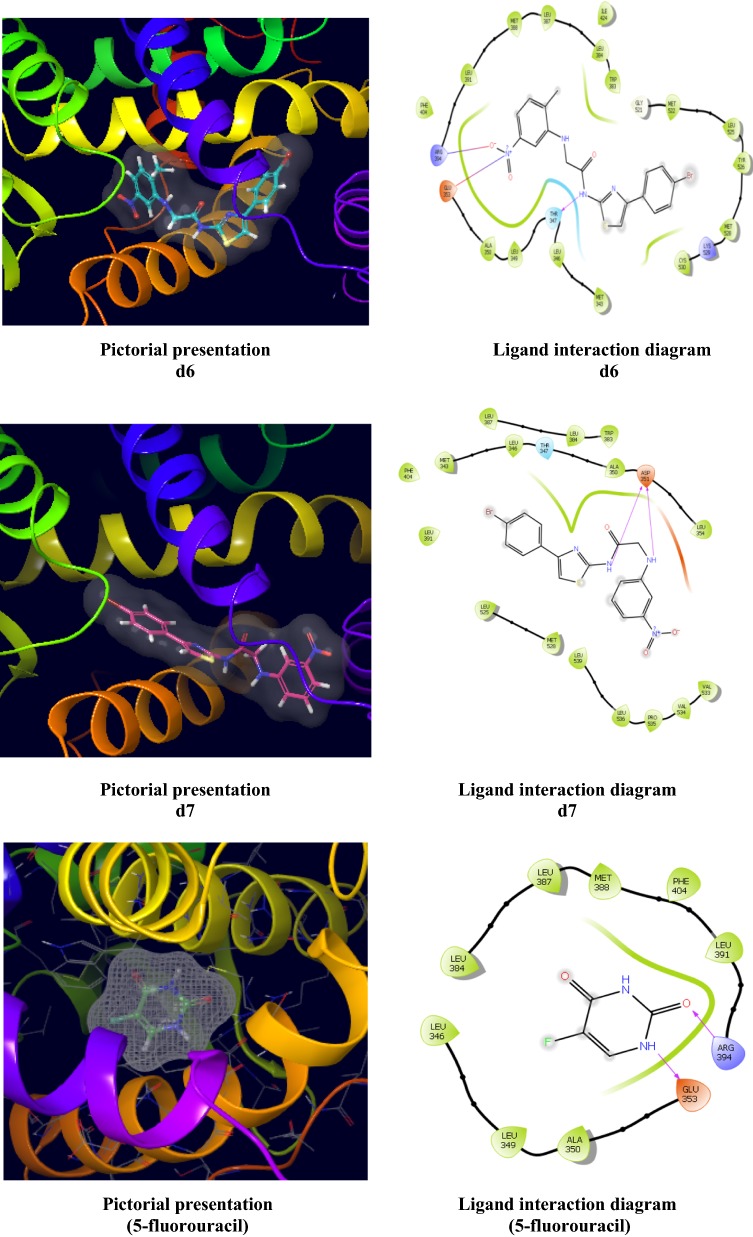
Pictorial presentation (3D) and Ligand interaction diagram (2D) of anticancer compounds (**d6** and **d7**) and 5-fluorouracil

The two most active anticancer compounds, **d6** and **d7** were docked in the binding pocket of ER-alpha of MCF7 (PDB ID-3ERT) co-crystallized with tamoxifen ligand. The results were examined based on docking score and glide energy values obtained by molecular docking study (Table 5, Fig. 7). The docking scores and glide energy values was illustrated in the negative terms. The more negative the docking score value, the better the binding affinity of ligand with the receptor. The ligand interaction diagram interpretated that nitro group of compound **d6** showed hydrogen bonding with the Glu353 and Arg394 amino acid residues and the nitrogen atom of the amide group formed hydrogen bonding with the Thr347 residue. The ligand interaction diagram interpretated that nitrogen of the amide group and nitrogen atom from aniline of compound **d7** formed hydrogen bonding with the Asp351 amino acid residue.

**Table 5:** Docking results of most active anticancer compounds (d6 and d7) with standard drug

Compounds	Docking score	Glide energy (kcal/mol)	Interactive amino acid residues
**d6**	− 7.353	− 50.355	Met343, Leu346, Thr347, Leu349, Ala350, Glu353, Arg394, Leu391, Met388, Leu387, Trp383, Gly521
**d7**	− 6.081	− 49.986	Met343, Leu346, Thr347, Ala350, Asp351, Leu354, Val533, Val534
**5-Fluorouracil**	− 3.414	− 24.58	Glu353, Ala350, Leu349, Leu346, Leu348, Leu387, Met388, Phe404, Leu391, Arg394

### Structure activity relationship study

**Fig. 8:**
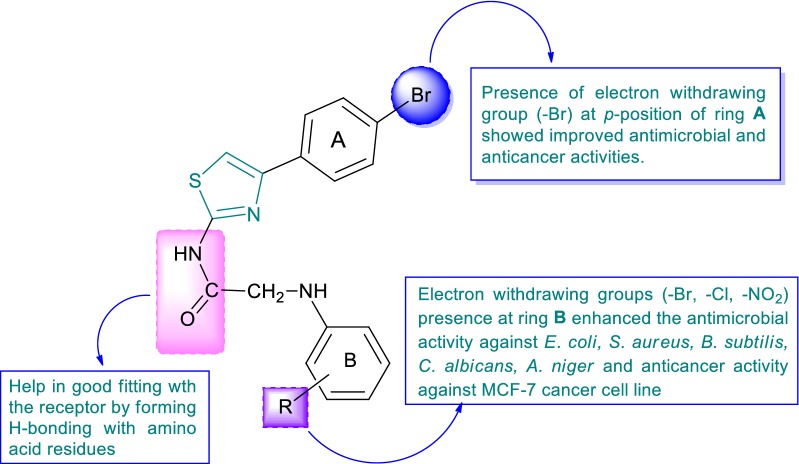
Structural activity relationship study of synthesized derivatives

The in vitro antimicrobial, antiproliferative and molecular docking results demonstrated the following structure activity relationship for *N*-(4-(4-bromophenyl)thiazol-2-yl)-2-chloroacetamide derivatives (Fig. 8):

- Electron withdrawing group (–Br) present at *para*-position of ring **A** improved the antimicrobial and anticancer activities of *N*-(4-(4-bromophenyl)thiazol-2-yl)-2-chloroacetamide derivatives
- The presence of electron withdrawing groups (–Br, –Cl, –NO_2_) at *ortho, meta* and *para*-position of ring **B**, improved antimicrobial activity against *E. coli, S. aureus,*
- *B. subtilis*, *C. albicans*, *A. niger* and anticancer activity against cancer cell line (MCF7).
- The presence of amide linkage with the synthesized compounds helps in H-bonding formation with the amino acid residues that leads to better fitting of compounds within the receptor and exhibited good antimicrobial and anticancer activities.

## Conclusion

In conclusion, synthesis, molecular docking and pharmacological potentials of new synthesized thiazole derivatives are presented in this paper. The synthesized compounds were evaluated for in vitro antimicrobial and antiproliferative activities against using microorganisms (bacterial and fungal) and cancer cell line (human breast adenocarcinoma). Among the synthesized derivatives, compounds **d1**, **d2** and **d3** showed promising antimicrobial activity and compounds **d6** and **d7** displayed better anticancer activity against human breast adenocarcinoma cancer cell line. Further, the molecular docking study indicated that compounds **d1**–**d3, d6** and **d7** showed the good docking score within binding pocket and comparable to the standard drugs. The docking results are consistent with the antimicrobial and cytotoxicity assays. Docking data remain in good correlation with antimicrobial and cytotoxic activity results and these active compounds may be used as a lead for rational drug designing.
